# The role of disulfide bond in hyperthermophilic endocellulase

**DOI:** 10.1007/s00792-013-0542-8

**Published:** 2013-04-27

**Authors:** Han-Woo Kim, Kazuhiko Ishikawa

**Affiliations:** 1 Division of Life Sciences, Korea Polar Research Institute (KOPRI), Incheon, 406-840 Korea; 2 Biomass Refinery Research Center, National Institute of Advanced Industrial Science and Technology (AIST), 3-11-32 Kagamiyama, Higashi-Hiroshima, Hiroshima 739-0046 Japan

**Keywords:** Disulfide bond, Cellulase, Archaea, Thermostability, Crystal structure, Protein engineering

## Abstract

The hyperthermophilic endocellulase, EGPh (glycosyl hydrolase family 5) from *Pyrococcus horikoshii* possesses 4 cysteine residues forming 2 disulfide bonds, as identified by structural analysis. One of the disulfide bonds is located at the proximal region of the active site in EGPh, which exhibits a distinct pattern from that of the thermophilic endocellulase EGAc (glycosyl hydrolase family 5) of *Acidothermus cellulolyticus* despite the structural similarity between the two endocellulases. The structural similarity between EGPh and EGAc suggests that EGPh possesses a structure suitable for changing the position of the disulfide bond corresponding to that in EGAc. Introduction of this alternative disulfide bond in EGPh, while removing the original disulfide bond, did not result in a loss of enzymatic activity but the EGPh was no longer hyperthermostable. These results suggest that the contribution of disulfide bond to hyperthermostability at temperature higher than 100 °C is restrictive, and that its impact is dependent on the specific structural environment of the hyperthermophilic proteins. The data suggest that the structural position and environment of the disulfide bond has a greater effect on high-temperature thermostability of the enzyme than on the potential energy of the dihedral angle that contributes to disulfide bond cleavage.

## Introduction

During the past decade, many researchers have attempted to develop an optimized enzyme that possesses high activity and stability suitable for industrial processes. Enzymes from hyperthermophiles have shown promising candidates for industrial applications because of their intrinsic thermal and chemical stability. The crystal structure of hyperthermophilic beta-1,4 endocellulases (EGPh, glycosyl hydrolase family 5, *T*_opt_ = 100 °C) from the archaeon *Pyrococcus horikoshii*, that was isolated from an oceanic hydrothermal volcanic vent, has been determined (Kim and Ishikawa 2010).

EGPh exhibits high structural similarity to the thermophilic beta-1,4 endocellulase (EGAc, glycosyl hydrolase family 5, *T*_opt_ = 81 °C) from *Acidothermus cellulolyticus.* Information regarding the structure of an enzyme greatly helps to understand its biophysical properties, such as thermostability. Typically, there are few disulfide bonds in hyperthermophilic proteins (Ladenstein and Ren 2008). Furthermore, disulfide bond stability might not be compatible with the high temperature of 100 °C (Volkin and Klibanov 1987). In the superimposed structure of EGPh and EGAc, the disulfide-bonding pattern of both enzymes was different despite their nearly identical structures. One of the disulfide bonds of EGPh is located at a region near the active site cleft, which is likely to be important for enzymatic activity, as well as for thermostability. Therefore, we investigated the functional role of the disulfide bond in EGPh using X-ray crystallographic and mutational analyses.

## Materials and methods

### Construction and preparation of the mutant enzymes

The EGPhΔN5C5 gene which is the truncated mutant gene lacking 5 amino-acid residues from the N- and C-terminal ends of EGPh was used as template for mutational analysis (Kim and Ishikawa 2010). Point mutant enzymes were prepared using site-directed mutagenesis following the Quick-Change Mutagenesis method (Stratagene, CA, USA). All mutant genes were inserted into the expression-vector pET11a (Novagen, Madison, WI, USA). The constructed plasmids were introduced into *Escherichia coli* strain BL21(DE3) for recombinant protein expression. Expression and purification of the recombinant enzymes was carried out following the method reported previously (Kim et al. 2007; Kim and Ishikawa 2011). The purity and molecular weight of the protein sample were analyzed by SDS-PAGE. The protein concentration of the enzyme was determined from UV absorbance at 280 nm, using 134,990 as the molar extinction coefficient calculated from their protein sequences, respectively.

### Activity measurement

The hydrolytic activity of the enzymes toward phosphoric acid swollen avicel (PSA) (Wako Pure Chemicals) was determined by measuring the amount of the released reducing sugars with the modified Somogyi-Nelson method (Hiromi and Ono 1963; Kim and Ishikawa 2011) at 85 °C in 100 mM Na/acetate buffer (pH 5.5).

### Crystallization

The purified proteins were dialyzed against 50 mM Tris–HCl buffer (pH 8.0) and then concentrated to 10 mg ml^−1^. Crystallization was performed using the hanging-drop vapor-diffusion method. The drops consisted of equal volumes (1.5 μl) of the protein and reservoir solutions. The crystal of the enzymes was prepared using a reservoir solution consisting of 1.5 M ammonium phosphate and 0.1 M MES buffer (pH 6.5). The crystals were obtained over a period of ~3 days at 22 °C.

### Data collection and processing

The crystals were collected with a Cryo-loop (Hampton Research, Aliso Viejo, CA, USA) and immediately flash-cooled at 100 K in a nitrogen cryostream. Diffraction data of the crystals were collected at BL44, SPring-8 (Harima, Hyogo, Japan), and processed and scaled using the HKL2000 and CCP4 package (CCP4 1994). The structure of the mutant enzyme was solved by molecular replacement using Molrep program implemented in CCP4 package. Refinement of the structures was performed with REFMAC5 in CCP4 package and Phenix.refine in the PHENIX package. All model building-stages were performed with the coot program (Emsley and Cowtan 2004). The diffraction data statistics and the crystallographic refinement statistics are summarized in Table 1. Figures were produced using PyMOL program (http://www.pymol.org).

**Table 1:** Statistics of data collection and refinement

	Wild type	P74C
Data collection		
Wavelength (Å)	0.9	0.9
Space group	*C121*	*C121*
Unit-cell parameters (Å)	*a* = 161.10, *b* = 58.34, *c* = 137.92, *β* = 109.65	*a* = 160.24, *b* = 58.68, *c* = 138.54, *β* = 108.93
Matthews coefficient (Å^c^ Da^−1^)	2.35	2.37
Solvent content (%)	47.68	48.16
Subunits per asymmetric unit	3	3
Resolution range (Å)	50–1.75 (1.81–1.75)	50–1.95 (1.98–1.95)
Number of observed reflections	457,155	385,303
Total number of unique reflections	121,695	89,041
Redundancy	3.8 (3.2)	4.4 (4.1)
<*I/*σ*(I)*>	37.9 (5.0)	20.3 (6.4)
*R*_merge_^a^	0.073 (0.434)	0.085 (0.305)
Completeness (%)	98.7 (87.5)	99.5 (98.5)
Refinement		
Resolution used in refinement	44.69–1.75 (1.77–1.75)	34.52–1.95 (1.97–1.95)
*R*_work_^b^ (%)	17.6 (27.4)	15.4 (15.3)
*R*_free_^c^ (%)	20.5 (31.6)	18.8 (22.7)
R.M.S. bond distance (Å)	0.007	0.007
R.M.S. bond angle (°)	1.072	1.083
Mean overall *B* factor (Å^b^)	23.99	15.61
Ramachandran plot		
In favored regions (%)	98.1	98.0
In disallowed regions (%)	0.0	0.0
PDB ID	3W6L	3W6M

Values for the last resolution shell are given in parentheses

^a^ *R*_merge_ = ∑_hkl_ ∑_*i*_ | *I*_*i*_(hkl) − <*I*(hkl)> | ∑_hkl_ ∑_*i*_ *I*_*i*_(hkl), where *I*_*i*_(hkl) is the *i*-th intensity measurement of reflection hkl, including symmetry-related reflections, and <*I*(hkl)> is their average

^b^ *R*_work_ = ∑_hkl_ | *F*_o_ − *F*_c_|/∑_hkl_ *F*_o_ where *F*_o_ and *F*_c_ are the observed and calculated structure factor amplitudes of reflection hkl, respectively

^c^ *R*_free_ is calculated as the *R*_cryst_, using *F*_o_ that was excluded from the refinement (5 % of the data)

### Differential scanning calorimetry

Differential scanning calorimetry (DSC) measurements were carried out using a nanoDSCII instrument (TA Instruments, DE, USA) with platinum tubing cells with a volume of 0.3 ml. Proteins were dialyzed against Na/acetate 50 mM (pH 5.5). The dialysate buffer was used as a reference solution for the DSC scanning. Samples containing 1.0 mg/ml of protein were heated at 1 °C/min from 5 to 125 °C.

## Results

### Structural comparison of disulfide bonds of EGPh and EGAc

**Fig. 1:**
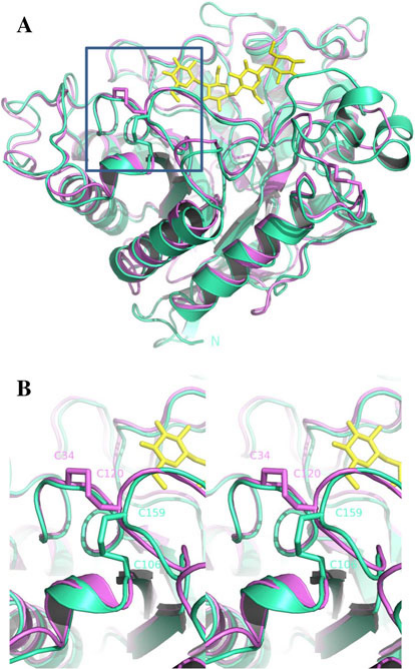
Comparison of the overall structure from *P. horikoshii* EGPh (*magenta*) and *A. cellulolyticus* EGAc (*light green*) (**a**) and close-up *cross-eyed stereo view* of the structure of the disulfide bonds in EGPh and EGAc (**b**). The cellotetraose from the EGAc-ligand complexed structure (PDB code: 1ECE) is denoted by *yellow color*

EGPh is a family 5 hyperthermophilic glycosyl hydrolase (*T*_opt_ = 100 °C). The crystal structure of EGPh revealed 4 Cys residues (C106, C159, C372, and C412) forming 2 disulfide bonds. The disulfide bonding of C106 and C159 forms at the end of the loop associated with the substrate-binding active site cleft (Fig. 1). The other bond appears to be located at the C-terminal region, although it could be not observed in the crystal structure owing to the absence of C412 in the truncated mutant used for protein crystallization. EGAc is a family 5 thermophilic glycosyl hydrolase (*T*_opt_ = 81 °C). Both enzymes belong to family 5, subfamily 1 cellulases (Kim and Ishikawa 2010), and resent sequence analysis has revealed putative disulfide-bonding Cys residues at the active cleft loop in a few homologs (data not shown). The superimposed structure of EGPh and EGAc demonstrates that C159 in EGPh is conserved in EGAc (C120) (Fig. 1b) but the pattern of the disulfide bond of EGPh (C106–C159) is different from that of EGAc (C34–C120) (Fig. 1).

### Construction and structural analysis of mutants

**Fig. 2:**
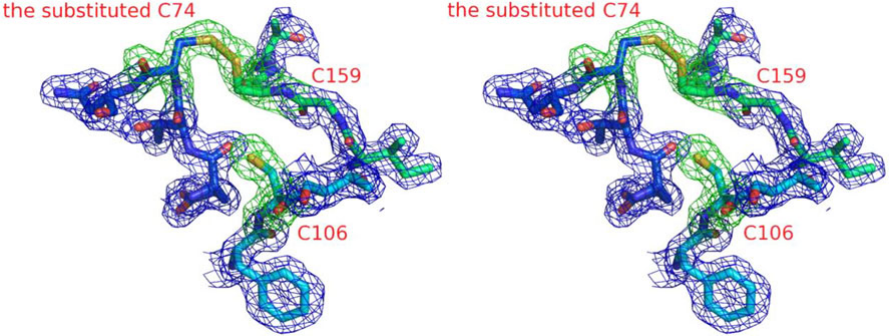
Cross-eyed stereo view of 2*F*_o_–*F*_c_ (*blue*) and *F*_o_–*F*_c_ (*green*) positive electron density in the omit map of P74C. The *F*_o_–*F*_c_ omit map (contour level: 3.0* σ*) was calculated prior to incorporation of 3 Cys residues in the structure model

**Fig. 3:**
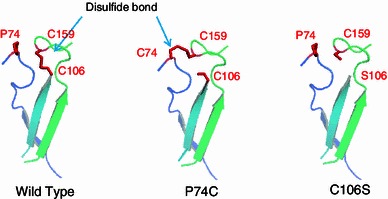
Comparison of the disulfide bonds from the mutants and wild type. P74C with 3 Cys residues showed an identical disulfide-bonding pattern to EGAc. C106S has no disulfide bond

The three-dimensional structural positions for the residues (P74, C106, C159) in EGPh are close to those for the residues (C34, S66, C120) in EGA (Fig. 1). However, the pattern of the disulfide bond of EGPh (C106–C159) is different from that of EGAc (C34–C120) (Fig. 1). Therefore, it is assumed that alternative disulfide bond can be introduced between P74 and C159 in EGPh. Given that the disulfide bond (C106–C159) is located too close to the active cleft in EGPh (Fig. 1b), we assumed that a conformational change of the region encompassing the disulfide bond might affect enzyme activity. To investigate a role of the disulfide bond in the activity and stability of the enzyme, we prepared 3 mutants of EGPh; P74C, C106S, and P74C/C106S, in which we removed the disulfide bond or changed the disulfide-bonding pattern. Based on the superimposed structures of EGPh and EGAc, the position of P74 in EGPh corresponding to C34 in EGAc involves the disulfide bond (C34–C120) in EGAc (Fig. 1b). Therefore, the position of P74 might be forming the disulfide bond to C159 in EGPh. The mutant C106S has only 1 Cys residue (C159); thus, disulfide bond cannot form around this site in EGPh. The mutant P74C/C106S possesses 2 Cys residues at the region and is expected to form a disulfide bond (C74–C159) structurally similar to that of EGAc (C34–C120). In the mutant P74C, we paid particular attention to which pair of residues among the 3 Cys residues (C74, C106, and C159) would participate in disulfide bond formation in EGPh. To visually confirm a disulfide bond in P74C, we prepared the P74C protein crystal using the condition described in “Materials and methods” and solved the crystal structure of P74C at a resolution of 1.95 Å (Table 1) (PDB ID: 3W6M). Figure 2 shows the structure model and the *F*_o_–*F*_c_ omit map around the 3 Cys residues in P74C. The structural data demonstrated that the substituted C74 residue formed a new disulfide bond (C74–C159) (Fig. 2). The newly formed disulfide bond (C74–C159) in EGPh was located at the conserved position for the disulfide bond (C34–C120) in EGAc (Fig. 1). The conformation of the newly formed disulfide bond in EGPh also exactly matched with that of EGAc on the superimposed structure. The neighboring residues around the Cys residues were not affected structurally by the mutation (Fig. 1). Moreover, as expected, the P74C/C106S mutation possesses an identical disulfide bond (C74–C159) conserved in EGAc. These results indicate that the formation of the disulfide bond at positions (C74–C159) is structurally stable in the folding process of EGPh. Figure 3 shows the comparison of the disulfide bonds from the mutants and wild type. The structure of P74C with 3 Cys residues obtained from the crystal structural analysis (PDB ID: 3W6M) showed an identical disulfide-bonding pattern to EGAc. The structure of C106S without disulfide bond (Fig. 3) was the model constructed from wild type. We calculated the potential energy of the disulfide bonds in the enzymes. In the disulfide bonds of the wild type and P74C, internal dihedral angles are approximately +90°, forming a typical right-handed hook conformation (Creighton 1988). Theoretical dihedral energies of disulfide configuration were calculated ignoring any possible influences of the residues around the disulfide bond. As shown in Table 2, the dihedral strain energy (DSE) (Schmidt et al. 2006) of C106–C159 in the wild type is slightly higher than that of C74–C159 in P74C.

**Table 2:** Comparison of the geometry and potential energy of the disulfide bond on the structures of wild type and the mutant P74C

Enzymes	S–S bond	Mol^a^	*χ*_1_	*χ*_2_	*χ*_3_	*χ*_2_′	*χ*_1_′	DSE (kJ mol^−1^)^b^	C_α_–C_α_ (Å)
WT	C106–C159	A	60.9	148.8	85.1	−89.8	−50.3	11.7	5.7
		B	58.0	150.4	84.3	−89.6	−52.5	10.9	5.7
		C	64.9	148.6	79.6	−90.4	−53.5	11.7	5.7
P74C	C74–C159	A	−58.9	−51.0	89.8	99.8	60.4	9.2	5.0
		B	−64.5	−46.1	88.3	91.9	62.2	8.3	5.1
		C	−55.9	−53.4	88.6	89.8	57.2	7.0	5.0

The five *χ* angles of the disulfide bond are represented as the below

^a^This protein crystal contains the 3 molecules in the symmetric unit

^b^The dihedral strain energy (DSE) of the disulfide was calculated using the following empirical formula (Schmidt et al. 2006)

DSE (kJ mol^−1^) = 8.37(1 + cos 3*χ*_1_) + 8.37(1 + cos 3*χ*_1_′) + 4.18(1 + cos 3*χ*_2_) + 4.18(1 + cos 3*χ*_2_′) + 14.64(1 + cos 2*χ*_3_) + 2.51(1 + cos 3*χ*_3_)

### Thermal analysis of the mutants

**Fig. 4:**
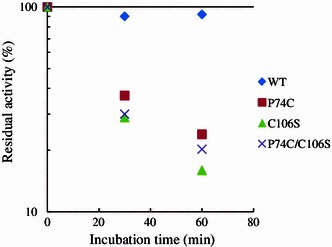
Thermostability of wild type and the mutants examined by measuring the residual activity after heat treatment. Heat treatment of the proteins (1.5 μM) was performed at 95 °C for each incubation time, and chilled immediately on ice. The residual activity was measured by detecting reducing sugar released from CMC

**Fig. 5:**
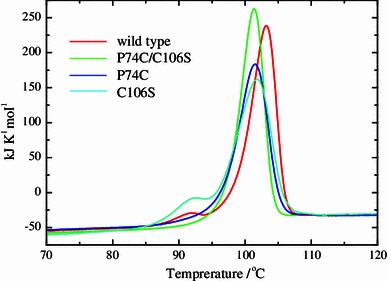
Differential scanning calorimetry (DSC) profiles of wild type and the mutants in 50 mM sodium acetate buffer (pH 5.5)

The mutants were examined for their activity using PSA. No significant change in the enzymatic activity at 80 °C was observed between the mutants and the wild type (data not shown), suggesting that the region involving the disulfide bond did not significantly influence substrate-binding or catalysis. To investigate the effects of disulfide bonds on irreversible thermostability, we determined the residual activity after heat treatment. As shown in Fig. 4, all of the mutants showed a dramatic decrease in residual activity after heat treatment compared with the wild type. Furthermore, we determined the melting temperature (*T*_m_) of the wild type and the mutant enzymes using DSC. The wild type enzyme was thermally unfolded at 103.4 °C. The melting temperature of the 3 mutants was approximately 101.5 °C (Fig. 5). These results indicate that the alternative new disulfide-bonding pattern involved in the position of P74 did not contribute to its hyperthermostability. We concluded, therefore, that only the disulfide-bonding pattern (C106–C159) in the wild type plays a role in enzyme stability in a hyperthermic environment.

## Discussion

Sequence analysis and structural comparison between EGPh and EGAc showed that they have distinct disulfide bond conformations in the variable region linked to the substrate-binding cleft, even though these two enzymes are both family 5 subfamily 1 cellulases. The Cys residues involved in disulfide bond formation are generally believed to be conserved among related homolog proteins (Overington et al. 1993; Richardson 1981). However, our recent analysis revealed that disulfide bond patterns are not necessarily conserved among homologues. Poorly conserved disulfide bonds were found mainly in the variable region, and they tend to stabilize the protein structure of the variable region (Rigden et al. 2011).

The crystal structural analysis of EGPh mutants revealed that a new disulfide bond at the position of P74 is formed in P74C and P74C/C106S. The structure of P74C with 3 Cys residues near the active site showed the same disulfide-bonding pattern as that of EGAc, suggesting that the conserved C159 prefers a linkage with the substituted C74 rather than with C106, unlike that of the wild type. Disulfide bond formation requires a strict stereochemical configuration. The angle between the sulfide and C_β_ atom of each Cys residue must be close to 103°, and the dihedral angle (*χ*_*3*_ in Table 2) of the 2 C_β_ atoms involved in disulfide bond must correspond to either +90° (right-handed) or −90° (left-handed) (Creighton 1988). In the structures of the wild type and P74C, the disulfide bonds have a near optimal geometry for the bond angle as well as the dihedral angle. When comparing redox potential, the disulfide bond in C74–C159 (P74C) possesses lower DSE than that in C106–C159 (wild type). The C_α_–C_α_ distance (5.0 Å) of C74–C159 was also slightly closer (5.7 Å) than that of C106–C159 (Table 2). These structural factors may allow the conserved residue C159 to easily form a disulfide bond with the substituted C74 rather than C106 of the wild type. Disulfide bonds with high potential energy are more easily cleaved than bonds with lower energy (Schmidt et al. 2006). However, the thermal analysis indicates that the C106–C159 bond with higher potential energy contributes to thermostability while the C74–C159 bond has no role in stability. This inconsistency and the stabilizing effect of the disulfide bond could be explained by the position of Cys residues. The residues C74 and C159 are located along flexible loop, and the position of C106 is located within a rigid region of strand structure (Fig. 3). Cross-linking via a disulfide bond with the rigid residue C106 may increase the rigidity of the flexible region involving the counterpart residue C159, resulting in increased conformational stability that affects thermostability (Fig. 3). The conformational differences in disulfide bonding between EGPh and EGAc imply that the *P. horikoshii* enzyme acquired (or retained) a disulfide bond conformation that increased its stability to extreme environmental conditions. The disulfide bond in the wild type plays a role in its stability on the hyperthermic environment. The introduction of the disulfide bond to the flexible region of the enzymes with higher DSE might be the strategy for maintain hyperthermostability of hyperthermophilic enzyme. The contribution of the disulfide bond toward thermostability in the wild type was not consistent with temperature over 100 °C, a temperature at which the host *P. horikoshii* survives, because the upper limit of disulfide bond stability has been believed to be 100 °C (Volkin and Klibanov 1987). This notion for susceptibility of disulfide bond is based on early studies to characterize protein inactivation mechanisms of mesophilic enzymes that were available at that time. Recent research on thermostable enzymes revealed that their disulfide bonds can protect against denaturation even above 100 °C (Cacciapuoti et al. 1994; Choi et al. 1999; Toth et al. 2000).

To improve the stability of an enzyme, one of the most commonly used technique in protein engineering is to introduce disulfide bond. However, only a few such cases have resulted in increased stability of the altered proteins relative to that of the wild-type protein (Han et al. 2009; Mansfeld et al. 1997; Mansfeld and Ulbrich-Hofmann 2007; Turunen et al. 2001). With regard to developing strategies for engineering disulfide bonds, it is important to recognize that a disulfide bond with a favorable position in a local structural environment may markedly increase thermal stability.
